# The Usability of a Smartphone-Based Fall Risk Assessment App for Adult Wheelchair Users: Observational Study

**DOI:** 10.2196/32453

**Published:** 2022-09-16

**Authors:** Mikaela Frechette, Jason Fanning, Katherine Hsieh, Laura Rice, Jacob Sosnoff

**Affiliations:** 1 Department of Kinesiology and Community Health University of Illinois at Urbana-Champaign Urbana, IL United States; 2 Siebel Center for Design University of Illinois at Urbana-Champaign Champaign, IL United States; 3 Department of Health and Exercise Science Wake Forest University Winston-Salem, NC United States; 4 Department of Internal Medicine Section on Gerontology and Geriatric Medicine Wake Forest School of Medicine Winston-Salem, NC United States; 5 Illinois Multiple Sclerosis Research Collaborative University of Illinois at Urbana-Champaign Urbana, IL United States; 6 Center on Health, Aging, and Disability University of Illinois at Urbana-Champaign Urbana, IL United States; 7 Department of Physical Therapy, Rehabilitation Science, and Athletic Training University of Kansas Medical Center Kansas City, KS United States

**Keywords:** usability testing, mobile health, wheeled device user, fall risk, telehealth, mHealth, mobile device, smartphone, health applications, older adults, elderly population, device usability

## Abstract

**Background:**

Individuals who use wheelchairs and scooters rarely undergo fall risk screening. Mobile health technology is a possible avenue to provide fall risk assessment. The promise of this approach is dependent upon its usability.

**Objective:**

We aimed to determine the usability of a fall risk mobile health app and identify key technology development insights for aging adults who use wheeled devices.

**Methods:**

Two rounds (with 5 participants in each round) of usability testing utilizing an iterative design-evaluation process were performed. Participants completed use of the custom-designed fall risk app, Steady-Wheels. To quantify fall risk, the app led participants through 12 demographic questions and 3 progressively more challenging seated balance tasks. Once completed, participants shared insights on the app’s usability through semistructured interviews and completion of the Systematic Usability Scale. Testing sessions were recorded and transcribed. Codes were identified within the transcriptions to create themes. Average Systematic Usability Scale scores were calculated for each round.

**Results:**

The first round of testing yielded 2 main themes: ease of use and flexibility of design. Systematic Usability Scale scores ranged from 72.5 to 97.5 with a mean score of 84.5 (SD 11.4). After modifications were made, the second round of testing yielded 2 new themes: app layout and clarity of instruction. Systematic Usability Scale scores improved in the second iteration and ranged from 87.5 to 97.5 with a mean score of 91.9 (SD 4.3).

**Conclusions:**

The mobile health app, Steady-Wheels, has excellent usability and the potential to provide adult wheeled device users with an easy-to-use, remote fall risk assessment tool. Characteristics that promoted usability were guided navigation, large text and radio buttons, clear and brief instructions accompanied by representative illustrations, and simple error recovery. Intuitive fall risk reporting was achieved through the presentation of a single number located on a color-coordinated continuum that delineated low, medium, and high risk.

## Introduction

Over 3 million individuals in the United States require the use of a wheelchair for mobility [1] and wheelchair use is expected to increase [2,3]. Although wheeled device use has numerous benefits [4], it presents several unique risks, such as falls. Roughly 75% of wheelchair users fall at least once a year [5-8]. Approximately 50% of reported falls cause injuries [7], ranging from minor (ie, abrasions) to serious (eg, fractures) [6]. Falls can also induce fear of falling [9] and activity curtailment [7], which are associated with isolation and decreased independence and quality of life [10].

Falls are detrimental to wheeled device users’ health and well-being, making fall risk screening a necessary part of overall health care. Although the US Centers for Disease Control and Prevention recommends annual fall risk screening for older adults, current screening recommendations are designed for ambulatory adults [11]. Moreover, fall risk screening is rarely performed in clinical practice, and there are numerous barriers to the implementation of effective fall prevention programs for wheelchair users. As a result, most individuals who rely on wheeled mobility do not undergo routine fall risk screening. Additionally, the COVID-19 pandemic and its related restrictions necessitate remote monitoring of health. This highlights the need for novel remote fall risk technology specific to this population.

Due to limited access, researchers are exploring innovative approaches to deliver comprehensive and objective fall risk assessment to wheeled device users. One possible method leverages the capabilities of smartphone technology by developing an at-home fall risk health app [12-16]. This approach has been examined in ambulatory adults with a range of physical function [17,18]. Building on this potential, it has been demonstrated that a smartphone-based approach is a valid and reliable method to distinguish wheeled device users with and without impaired seated postural control [19]. Collectively, these findings provide the rationale for the development of an objective mobile health app that can provide wheeled device users with at-home fall risk assessment.

Although there is a strong rationale for the development of this type of health app, ensuring that such a tool is easy to use and provides intuitive fall risk score reporting is a necessary precursor to its future use in health behavior interventions [20]. Consequently, the purpose of the current study is to determine the usability of a fall risk mobile health app, Steady-Wheels, and identify key technology development insights for aging adults who use wheeled devices. This health app is an adaptation of a pre-existing fall risk app for older adults [18]. Based on prior investigations, we hypothesized that this health app would have a high level of usability.

## Methods

### Underlying Design Considerations

When designing the first iteration of the health app, we considered our target users’ (individuals aging with a physical disability) characteristics (Table 1). To ensure a high degree of usability, age-related changes and limitations due to disease or injury were taken into consideration, particularly as they related to cognitive overload, dexterity, and sensory function. To reduce cognitive overload, the app was designed to provide written instructions immediately preceding a task. Only one set of instructions was presented per slide, and large graphics depicting the task were also provided. This layout streamlined the app and reduced the need for working memory of the participants. In total, there were 14 slides, taking approximately 10 minutes to complete. Decrements in dexterity are commonly seen in those who have neurological complications [21-23] and age-associated arthritis [24]. To account for this within the app, selection options and buttons were made large, and typed responses were avoided. Sensory-related changes were accommodated by the use of black text written in a 14-point font on a white background [25], and auditory processing deficits were accommodated by the use of leading audio cues with simultaneous vibrations.

**Table 1:** Participant demographic information.

Characteristics			First iteration		Second iteration
Age (years), mean (SD)			59.0 (12.2)		58.0 (13.1)
**Sex, n (%)**					
	Male	3 (60)		2 (40)	
	Female	2 (40)		3 (60)	
Smartphone usage, n (%)			5 (100)		5 (100)
Time using mobility device (years), mean (SD)			25 (20.3)		25 (27.4)
**Primary mobility device, n (%)**					
	Power chair	4 (80)		2 (40)	
	Manual chair	1 (20)		0	
	Scooter	0		3 (60)	
**Reason for wheeled mobility, n (%)**					
	Multiple sclerosis	2 (40)		4 (80)	
	Paraplegia/quadriplegia	2 (40)		1 (20)	
	Stroke	1 (20)		0	
History of falls (≥1 falls/year), n (%)			1 (20)		2 (40)
Self-reported fear of falling, n (%)			5 (100)		5 (100)
**Level of education, n (%)**					
	High school graduate/General Educational Development Test Credential	0		1 (20)	
	Some or in-progress college/associate degree	2 (40)		0	
	Bachelor’s degree	0		1 (20)	
	Master’s degree	3 (60)		2 (40)	
	Doctoral degree	0		1 (20)	

### Components of the Steady-Wheels App

**Figure 1:**
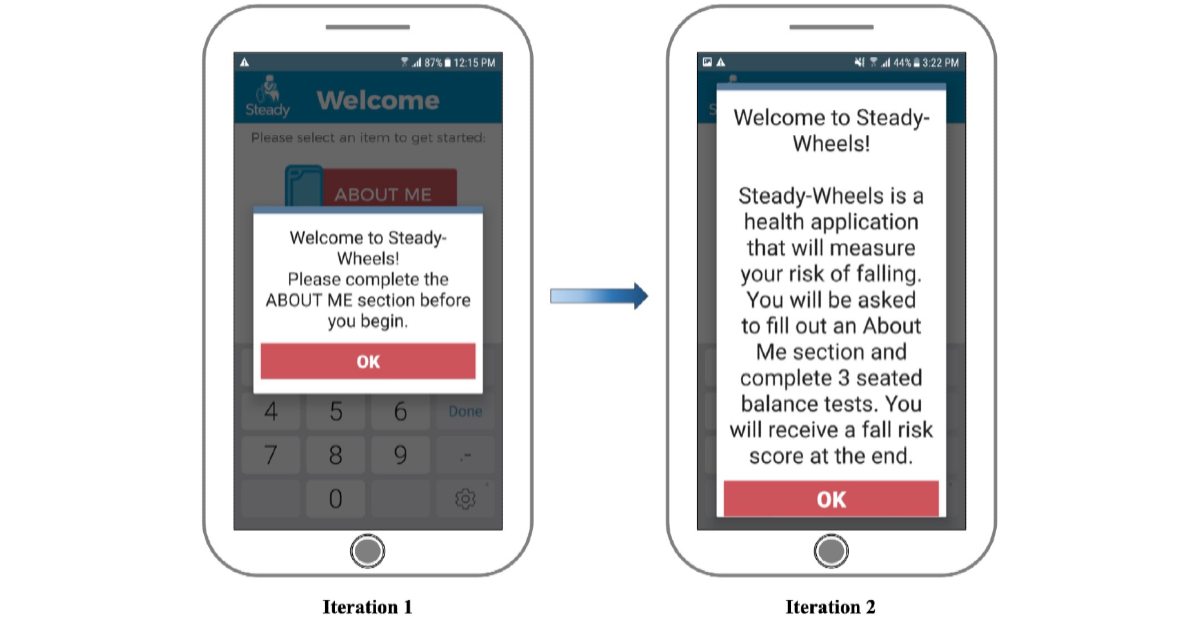
The text size was increased and the content was modified from iteration 1 to iteration 2 to allow for greater ease of use.

**Figure 2:**
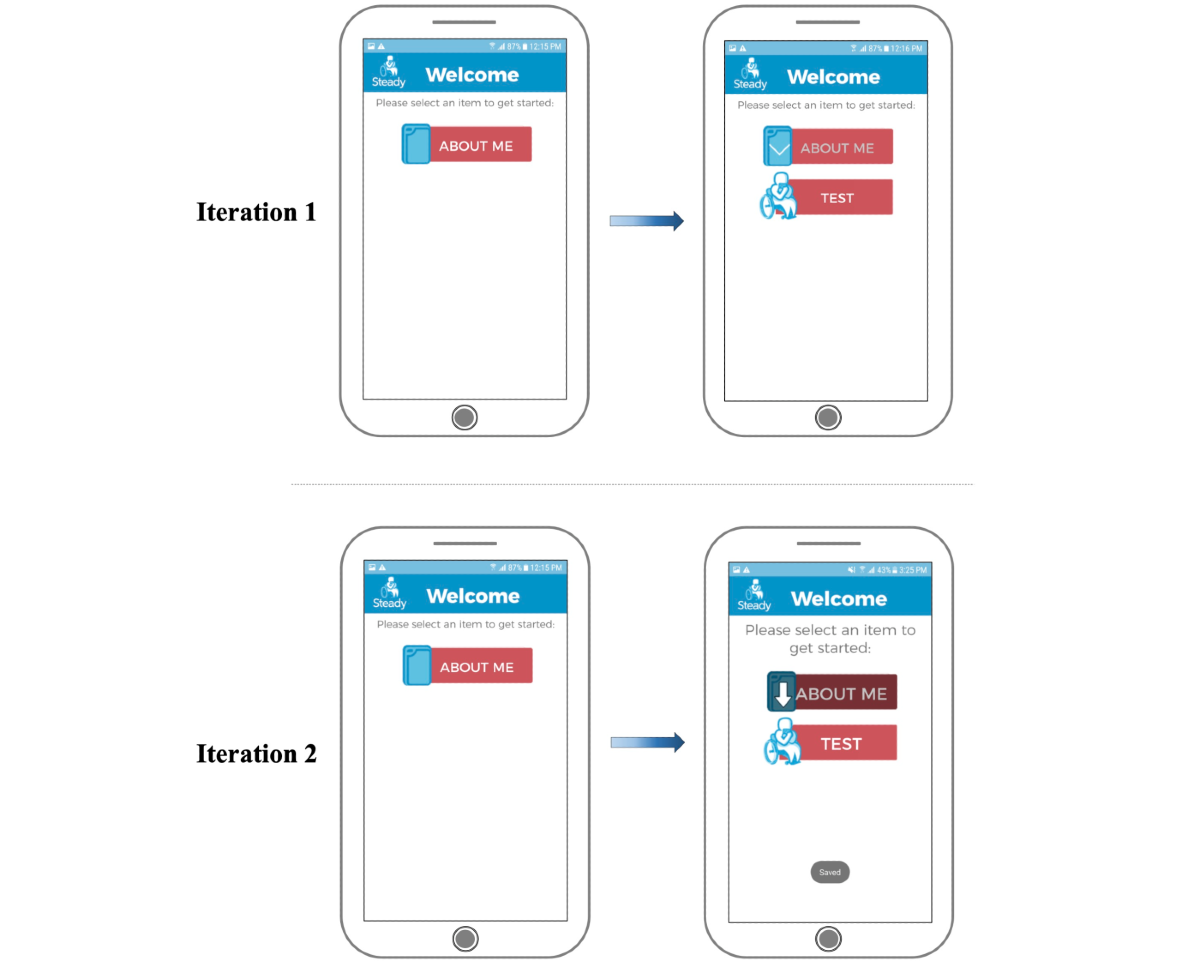
Onscreen instructions were enhanced from iteration 1 to iteration 2.

**Figure 3:**
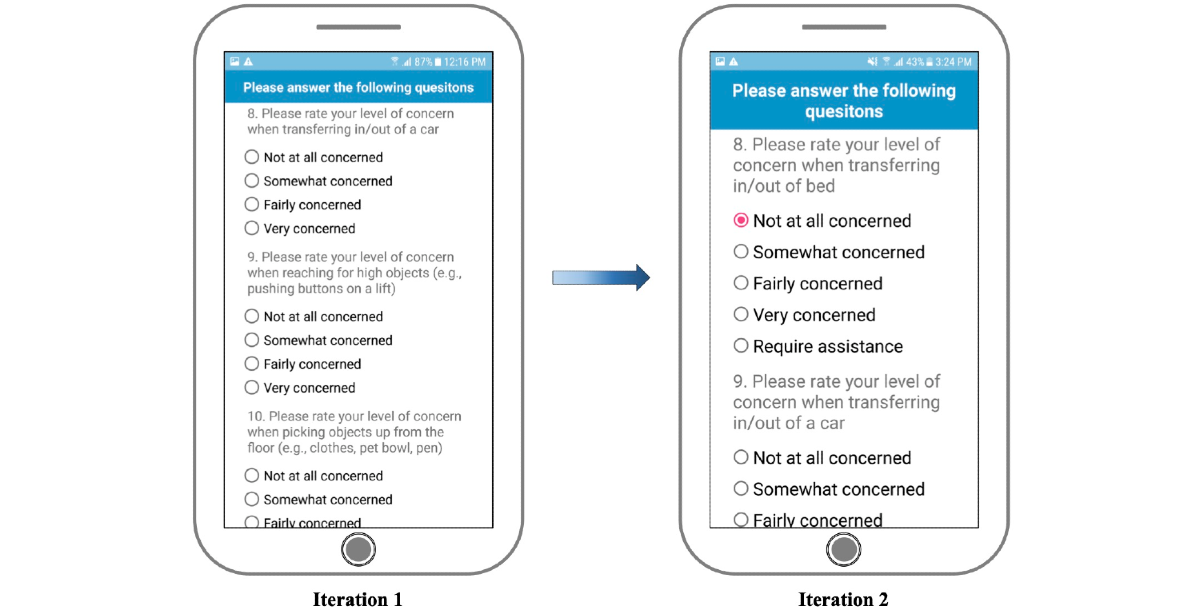
Changes made from iteration 1 to iteration 2 within the “About Me” section included larger text, larger radio buttons, and more choice response options.

**Figure 4:**
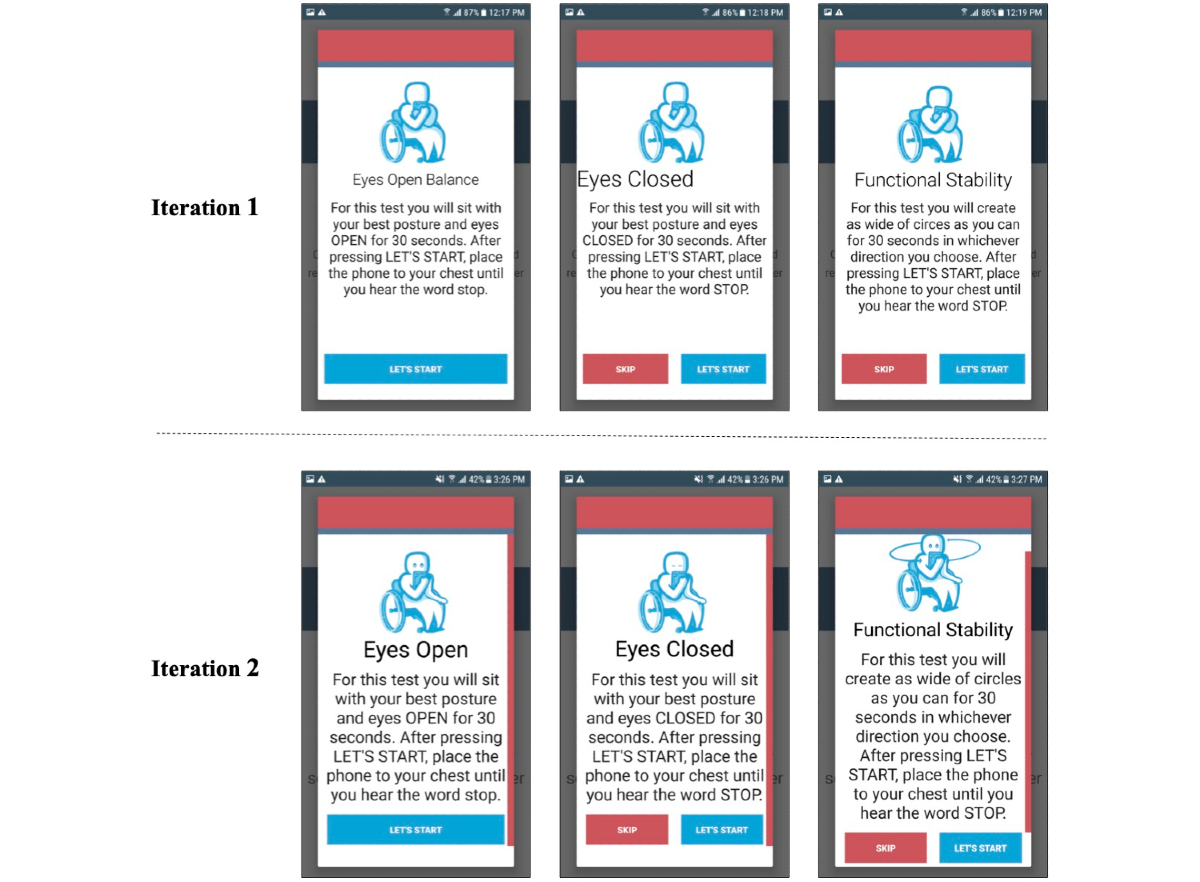
Modifications were made to the onscreen balance task instructions from iteration 1 to iteration 2.

The fall risk app, Steady-Wheels, was developed in Android Studio 3.1.2. Upon opening the app, users are presented with a welcome screen that outlines the purpose of the app and provides an overview of the process (Figure 1). Steady-Wheels has two main components: a patient-reported outcome section and a performance test section (Figure 2). The patient-reported outcome component asks the participant to complete a 13-item health history questionnaire (including age, sex, number of falls in the last year, and activities that provoke concerns about falling [9]) (Figure 3). The performance component leads participants through a progressive series of seated postural control tasks (Figure 4). Before testing, participants were provided with written safety instructions. Participants were instructed to engage their wheel locks, and power wheelchair and scooter users were instructed to turn off their devices. All participants were asked to have a handrail or wall nearby in case they lost their balance. To complete the testing, the device guided participants through the completion of three 30-second seated balance tasks in a standardized order that increasingly challenged the participant’s base of support: an eyes-open balance task, an eyes-closed balance task, and a functional stability boundary task (Figure 4). These tests were chosen because they can provide insight into postural control [26] and have been linked to fall risk [27]. Written instructions on how to properly complete the balance tasks were provided before the start of each task. After the participant self-selected the “Let’s Start” option, an audio and vibratory countdown began from 5, leading to the word “start,” which cued the start of the test. The completion of the test was auditorily cued with the word “stop.” Participants were asked to hold the smartphone against the middle of their chest with their dominant hand for the duration of each test. Upon completion of each balance task, users reported if they were able to complete the task by selecting one of the following: “I completed the test,” “I was unable to complete the test,” and “I did NOT attempt to complete the test.” If participants were dissatisfied with their attempt at the task, they could select “I’d like to retry” and make another attempt.

**Figure 5:**
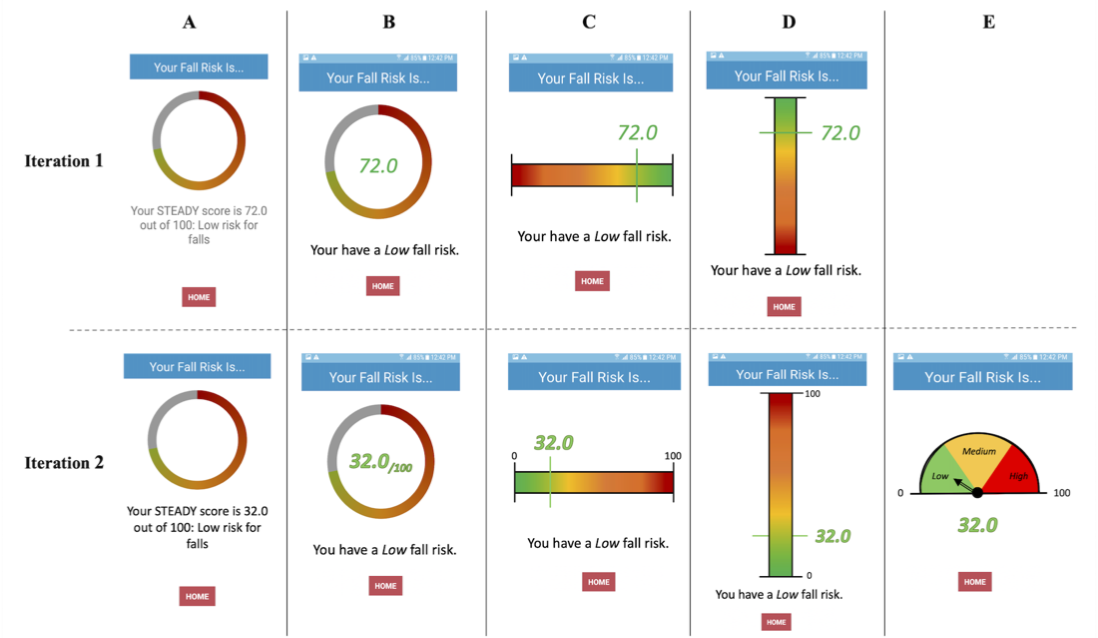
Different options for the result screen (A-E), with modifications made from iteration 1 to iteration 2.

A future goal of this work is to utilize the participants’ demographic and movement data to generate a personalized fall risk score. To better understand users’ preferences for receiving their fall risk score, they were asked to rate different result screen options and provide insight on what made some illustrations better than others (Figure 5).

### Ethics Approval

The Institutional Review Board of the University of Illinois at Urbana-Champaign approved all procedures (20192), and all participants provided informed consent before engaging in research activities. All research procedures were performed in accordance with the ethical standards of the responsible committee on human experimentation (institutional and national) and with the Helsinki Declaration of 1975.

### Participant Characteristics

To be eligible, individuals were required to be ≥18 years old, utilize a wheeled mobility device for their main form of mobility, be able to sit unsupported for 30 seconds, have manual dexterity sufficient to use a smartphone, have hearing and vision that were normal or corrected to normal, and be able to read and speak English. In light of the COVID-19 pandemic, having access to video conferencing software (eg, Zoom, Facetime, or Skype), was an inclusion criterion for the second round of testing.

This study included 2 rounds of 5 different older adult wheelchair users (age 58.5 years, SD 12.6 years; 5 male, 5 female) who were recruited from the community through existing participant pools, sharing of research flyers, and word of mouth (Table 1). The first round of testing was completed in person between November 2020 and February 2021, while the second round was performed remotely between April and May 2021. During sessions, participants completed using the app, identified barriers to usability, and gave their rationale for their preferred results options. Feedback from the first round of 5 participants was used to modify the app. Following modification, the second round of volunteers participated in usability testing. This iterative design process is ideal for identifying use challenges, and having a sample size of 5 individuals per round of testing has been shown to be sufficient for identifying usability problems [28,29]. On average, the first round of testing in iterative design identifies 85% of usability problems, and the second round identifies an additional 13% [30]. This approach has been successful in the development of various health apps [31-33], including 2 recent fall risk apps for older adults [18] and patients with multiple sclerosis [34].

### Experimental Session

After providing informed consent, each participant was given a smartphone (Samsung Galaxy S6, Samsung) that had the Steady-Wheels app installed. The participants were read an instructional prompt (Multimedia Appendix 1) asking them to speak their thought processes aloud while they independently used the app [35]. After the questions were answered, the researchers began visually and auditorily recording the participants’ interactions with the app and wrote field notes. After they completed using the app, the participants completed a semistructured interview in which they were asked to expand upon their likes and dislikes about the app’s layout and features (eg, graphics and wording) and to provide any suggestions for future iterations of the app. During this time, the participants also ranked the fall risk score results options from most to least favorite (Figure 5).

For the most part, these procedures remained constant for the second round of usability testing. The only difference was that the research supplies were delivered to the participants’ residences and the experimental session was completed over video conferencing software.

Along with feedback from the participant interviews, a smartphone usage questionnaire and the System Usability Scale (SUS) [36] were used to understand the participants’ experiences using smartphone and health apps and to quantify the usability of Steady-Wheels, respectively. While the questionnaire had a total of 6 choice and written response questions, the SUS consists of 10 questions with 5 response options [36,37], ranging from “strongly agree” (5 points) to “strongly disagree” (1 point). After calculation, results from the SUS range from zero (lowest usability) to 100 (highest usability); technology in general has an average score of 60 [37].

### Qualitative Analysis

A thematic analysis approach was used to conduct the qualitative analysis [38]. Video recordings from the think-aloud activity and interviews were transcribed verbatim. The text was then independently reviewed and assigned codes (eg, instructions, testing duration, and graphics) based on its content using the software MAXQDA (version 12.3.3; Verbi GMBH). Once codes were reviewed and discussed by 2 authors (MF and KH), they were grouped into themes based on the commonality of the data. The same 2 authors (MF and KH) then deliberated on the main themes to ensure they reflected participant insights as accurately as possible. Both authors had prior experience conducting qualitative analyses.

## Results

Participant demographic information is provided in Table 1. Table 2 presents participant responses as the mean response score (with SD) to each question of the SUS for the first and second iterations.

**Table 2:** Participant responses to each System Usability Scale question for the first and second iterations.

System Usability Scale question	Prompt	First iteration, mean score (SD)	Second iteration, mean score (SD)
1	I think that I would like to use this app frequently.	2.6 (1.7)	3.0 (1.6)
2	I found the app unnecessarily complex.	1.0 (0)	1.0 (0)
3	I thought the app was easy to use.	4.8 (0.4)	4.8 (0.5)
4	I think that I would need the support of a technical person to be able to use this app.	1.3 (1.3)	1.0 (0)
5	I found the various functions in this app were well-integrated.	4.2 (0.8)	4.5 (0.6)
6	I thought there was too much inconsistency in this app.	1.4 (0.5)	1.0 (0)
7	I would imagine that most people would learn to use this app very quickly.	4.8 (0.4)	4.8 (0.5)
8	I found the app very cumbersome to use.	1.2 (0.4)	1.0 (0)
9	I felt very confident using the app.	4.4 (0.5)	5.0 (0)
10	I needed to learn a lot of things before I could get going with this app.	2.0 (1.4)	1.3 (0.5)

### Iteration 1

The first round of usability testing yielded 2 themes: ease of use and flexibility of design. Representative participant quotes concerning these themes are reported throughout the following sections. The quotes are accompanied by participant characteristics (eg, sex, and age). System usability scores ranged from 72.5 to 97.5 and averaged 84.5 (SD 11.4), indicating “excellent” usability [39].

#### Ease of Use

Some participants found the app easy to use, saying it was “...very, very straightforward, very easy. I don’t happen to have much to say because it’s pretty straightforward” (male, 47 years old). Others had difficulty determining the sequence in which to complete the separate modules, stating, “There's only one item here. It says about me. Is that what I’m supposed to touch?” (male, 72 years old); this module can be seen in Figure 2. Following the completion of the “About Me” section, another participant said, “Now do I do the test?” (female, 43 years old). Although most participants were able to navigate the app, their thought processes indicated unnecessary cognitive load regarding the app layout: “Okay. Now we're ready to do the test portion I assume since I filled out the about me, so I’ll go ahead and do that” (male, 47 years old). Such insights may help to explain the large variance in participant responses to SUS question 10, which asks “I needed to learn a lot of things before I could get going with this app” (Table 2). In response to this feedback, the welcome screen was edited to provide a more thorough description of what the app was going to ask of the participant and the order in which it would be completed (Figure 1). The transition from the “About Me” section to the “Test” section was made more evident by shading the completed “About Me” option and providing a larger arrow pointing to the “Test” option (Figure 2).

Further participant feedback indicated that the app could be improved by having larger text and multiple-choice buttons, particularly within the “About Me” section. One participant said, “The layout? I guess I would say that some of it is a little bit small in terms of text and radio buttons. Since you're really focusing on your design you could blow it up a little...there's plenty of real estate to play with, so you might as well. Especially given the demographics of the people that will be using it—easier to make it more accessible” (male, 47 years old). Figure 1, Figure 3, and Figure 4 illustrate the changes made to text and radio button size. This increased font size led to the introduction of a vertical slide bar on slides that no longer fit on a single screen (Figure 4). Seated balance task titles were bolded and centered to draw attention (Figure 4).

The purpose of the app’s graphics was to help users further understand the instructional text. Based on participant feedback, it became apparent that the graphics used within the first iteration could be further refined. One participant stated, “It'd be easier if it [the graphics] demonstrated exactly what it was saying” (male, 47 years old). To better depict the nature of the tasks, open and closed eyes were added, an arm was moved to the side of the icon’s body to illustrate that only one hand was needed to hold the phone to the chest, and circular arrows were positioned around the icon completing the functional stability boundary task to represent the movement pattern of the task (Figure 4).

#### Flexibility of Design

Steady-Wheels aims to be applicable to all wheeled device users, but many participants showed difficulty answering the demographic questions accurately, due to the limited choice response options. Participants said, “Level of concern when reaching for higher objects? Well, I would normally ask for help” (male, 72 years old) and “Please rate your level of concern when pushing a wheelchair on uneven surfaces. Well, I don't push my wheelchair anymore” (male, 72 years old). The limited choice response options may have led participants to feel as if the app was not tailored to them, leading to the large variance in participant responses to SUS question 1, which asks, “I think that I would like to use this app frequently” (Table 2). To be more inclusive and comprehensive, more choice response options, such as “require assistance” and “I use a powered device” (Figure 3) were added, in addition to another demographic question asking, “What mobility device do you most commonly use?” with response options of “power wheelchair” or “manual wheelchair.”

App features that support individual preferences and allow for easy recovery from errors are known to increase the usability of a system. One feature that participants enjoyed was being able to swipe right to left to progress through the slides and left to right to retrieve prior slides. One participant said “Swiping works. That's useful. In addition to the buttons [eg, “next,” “back,” and “skip”], swiping left or right seems to work fine” (male, 47 years old). In addition to this, participants had the flexibility to change multiple-choice responses, retrieve prior slides, and reassess balance tasks if they were not pleased with their performance. Another participant said, “Whoops, can I go back? I missed something. It asked me a question” (male, 72 years old). For this participant, having the ability to retrieve prior slides and add or adjust their responses to questions was necessary for the accurate completion of the app. This flexibility of use also helped to counterbalance the difficulties associated with small radio buttons, which we have already discussed.

Primary modifications to the app were to increase the size of text and radio buttons for multiple-choice responses (Figure 1, Figure 3, and Figure 4), improve the on-screen directions (Figure 2 and Figure 4), and add response options to the demographic questions (Figure 3).

#### Preferred Results Screens

During the first iteration, participants strongly favored the result screen that showed a horizontal scale, numbered from 0 to 100 (Figure 5 C). Positive attributes of this option were the color scheme, the large fall risk number, the brief description of fall risk level, and the horizontal layout. Some criticisms included the lack of upper and lower bounds on the scale, (eg, “72 out of what?” [male, 72 years old]) and lack of clear low, medium, and high cut-off locations on the sliding scale. The participants also felt that a lower fall risk should be represented by a lower number. This feedback informed the development of new results screen options for the second iteration of testing.

### Iteration 2

The second round of usability testing yielded 2 themes: app layout and clarity of instruction. SUS scores ranged from 87.5 to 97.5 and averaged 91.9 (SD 4.3), indicating “best imaginable” usability [39].

#### App Layout

In general, participants were very pleased with the layout of the app during the second iteration of testing. One stated, “I think overall, it's good. I think it's clear” (female, 52 years old) and “I think it was straightforward...it was rather clear, concise, and pretty compact” (male, 42 years old). These improvements may help explain the minimal variance in participant responses to SUS question 10: “I needed to learn a lot of things before I could get going on this app” (Table 2). Although there were no clear modifications that needed to be made to the layout, one participant provided some insight into the app’s instructions by stating, “It was all very user friendly, self-explanatory, if you take the time to read it.” (male, 53 years old). This statement suggests that the app had high-quality instructions, but perhaps too many of them. Further synthesis of the instructions or the inclusion of visual aids may help alleviate this in future iterations.

#### Clarity of Instructions

The only instructions that received criticism were the ones for the functional stability boundary test. Despite changes to the visual representation (Figure 4), most participants struggled to understand how to complete the test. One participant said, “Well, I don't know what to do with this one. It says, ‘create as wide of circles.’ I don't know if that's with my wheelchair, in which case, I’d have to turn it back on. And if I do, I can't hold the phone to my chest.” (female, 71 years old). For clarity, the instructional text will be altered to read “For this test, you will create as wide of circles with your trunk as you can...”

#### Preferred Results Screens

During the second iteration, participants strongly favored the result screen option that showed a dial (Figure 5 E), stating that it was a “real obvious one,” and complimenting its representation of low, medium, and high risk. Many related it to their preexisting understanding of a speedometer.

During both iterations, participants enjoyed the simplicity of receiving a single score, stating, “I think having a clear and concise one or two number metric is great. That's perfect” (male, 42 years old). However, most had lingering questions, such as “How do I use this number?” (male, 72 years old) and “What does it tell me?” after the app was complete. “Maybe give some more information about how the score is actually generated, and maybe give some feedback...maybe having a pop-up recommendation screen at the end for some suggestions with exercises or something like that, might be utilitarian” (male, 42 years old). Further changes to the app are needed to investigate how much information is appropriate and informative for users.

## Discussion

### Principal Results

Understanding the usability of a smartphone app provides insight into the quality and overall satisfaction of the user’s experience. An improved experience could lead to greater use of health apps and increased adherence to suggested interventions [40]. Consequently, the purpose of the current study was to determine the usability of a fall risk mobile health app, Steady-Wheels, and identify key insights into technology development for aging adults who use wheeled devices. Initial design considerations were based on age-related changes and physical limitations associated with disability, including motor, sensation, and cognitive impairments. A mixed-method, iterative design and testing process yielded high SUS scores; the app was rated as having “excellent” and “best imaginable” levels of usability. The main themes for each iteration were informed by participant feedback, with the first round of testing yielding 2 main themes (ease of use and flexibility of design) and the second round of testing yielding 2 different main themes (app layout and clarity of instruction). These themes helped identify insights into app development that could promote usability for aging adults who use wheeled devices.

Overall, participants found that the app was straightforward, easy to use, supportive of individual preferences, and allowed for easy recovery from errors. They appreciated the simple, objective fall risk score. App development and modifications came from participant feedback and insights from previously developed apps [18,34,40-42] and an understanding of usability heuristics for interface design, such as the visibility of the system, use of recognition rather than recall, aesthetics, minimalist design, error prevention, and a match between the system and the real world [43].

The visibility of the system and the timeliness and adequacy of feedback and information to users informed the modifications made to the welcome screen and the overall “step-by-step” style of the app. This approach allowed for the recognition of a recently described task rather than recall of prior instruction. While this promoted the ease of use of Steady-Wheels, the primary complaints about health apps made by users are often related to their aesthetics, especially poor or difficult to interpret color coding, graphics, and fonts [42]. Providing a simple color scheme and font with the inclusion of only essential graphics aided this and created minimal distractions within the app. This approach placed a reduced cognitive load on users and helped to reduce the occurrence of errors.

The primary goal of the results scores at the end of testing was to intuitively convey fall risk results to diverse users. Individuals learn and retain content better from visual information (eg, cartoons and graphics) [44] and can interpret its meaning much more easily if the design is recognizable [41] or matches real-world experiences and expectations [43]. Providing users with a results option that mimicked their preexisting knowledge of speedometers followed these concepts, was well received, and promoted curiosity in the users about what could be done to lower their fall risk. Collectively, these design features led to the development of an app with high perceived ease of use, which is associated with greater adoption of technology [45].

Along with the personal adoption of technology, it is also important to gain insights into the likelihood of users recommending this technology to other individuals. The SUS has a strong relationship with the Net Promotor Score, which has become a common metric to understand customer loyalty [46]. Consumers will likely promote a product if it achieves a SUS score of 81 or greater [39]. In the current study, both iterations of testing yielded a SUS score above this threshold. This is particularly noteworthy as technology in general has an average SUS score of 60 [37]. Overall, these findings indicate that Steady-Wheels may not only be adopted on a personal level, but will likely also be recommended to others at an equal or greater rate than other forms of technology.

### Lessons Learned

Although this is the first app designed to measure fall risk in aging adults who use wheeled devices, our initial design was informed by the needs of users and learned experiences from prior attempts to develop fall risk screening apps, both for older adults [18] and for people with multiple sclerosis [34]; all of which have received high scores for usability from their respective users. Throughout the iterative testing of Steady-Wheels, we identified key insights that could further inform the development of mobile health apps for older users of wheeled devices. Depending on their physical ability, some individuals are reliant on the use of a single hand for all activities of daily living. Future development of remote assessment should account for this in the test selection and the test’s method of completion. By failing to consider this, researchers may increase the task’s safety risk, complexity, and error rates. It was common to see participants that had difficulties with dexterity, making large buttons to select and swiping options key to the app’s ease of use. Also critical to ease of use was the guided (step-by-step) navigation of the app with clear and brief instructions accompanied by representative illustrations along the way. These features helped to reduce the risk of errors, but if mistakes were made during testing, simple error recovery (eg, allowing for retests or access to previous slides to adjust choice responses) should be made possible. Lastly, intuitive fall risk reporting was achieved through the presentation of a single number located on a color-coordinated continuum for low, medium, and high fall risk. While participants found the simple reporting of their fall risk score to be useful, they were eager to learn ways to improve their fall risk. Providing follow-up preventative information may increase the app’s usefulness and encourage further engagement with the app and shared content [40]. Personalized messaging is an easy and effective strategy for altering patient behavior [47] and could be a feasible way to share such information.

Due to the COVD-19 pandemic, the second iteration of this study was completed remotely. This successful experience highlighted the potential feasibility of the home use of the app. Participants received a smartphone that they may not have been previously exposed to, but were able to turn it on, locate the previously installed app, and follow the instructions to completion. The validity and reliability of this novel measurement tool will need to be measured and compared to common clinical tests [48,49].

### Limitations

The current investigation has three primary limitations: (1) baseline interviews with target users were not conducted to inform the app’s initial iteration, (2) all participants in the second round of usability testing were required to have access to videoconferencing software (eg, Zoom, Skype, or Facetime), and (3) 9 of the 10 participants had received some form of higher education.

While the current app considered our target users’ characteristics and abilities and the lessons learned during the development of previous fall risk apps, baseline interviews were not performed. Taking a more traditional user-centered approach would likely have highlighted additional thoughts, wants, and needs concerning technology and the app’s design. Identifying these key insights early on would have been a way to better serve the target users and help ensure that the researchers’ time and resources were being used most efficiently.

Although the second round of usability testing helped to provide insights into the app’s feasibility in a home setting, exclusively enrolling individuals that already used videoconferencing software may have created a biased, “technology-friendly” sample. Unfortunately, this was the only possible method of testing during the COVID-19 pandemic. Moving forward, researchers should aim to prioritize in-person data collection sessions when possible.

Despite efforts to recruit through a variety of methods and locations, most participants had received some form of higher education. Higher education may provide individuals with more experience engaging with technology and a better understanding of it, and our participants may have been more likely to understand the fall risk scores as presented. This, too, may have contributed to bias. Future researchers should consider accounting for this effect by enrolling roughly equal proportions of individuals with different educational backgrounds.

### Conclusions

Previous literature has demonstrated that falls are common for individuals who use wheeled devices and are detrimental. The development of an objective, remote fall risk assessment tool could allow for accessible fall risk screening. Smartphone technology is a promising way to provide users with this information. Overall, aging adults who use wheeled devices found the mobile health app easy to use with a high level of usability due to characteristics such as guided navigation of the app, large text and radio buttons, clear and brief instructions that were accompanied by representative illustrations, and simple error recovery. Intuitive fall risk reporting was achieved through the presentation of a single number on a continuum of colors indicating low, medium, and high risk. Future apps developed for fall risk reporting for this population should consider leveraging the insights identified here to maximize usability.

## Appendix

Multimedia Appendix 1 Instructional Prompt.

## Abbreviations

SUS: System Usability Scale
