# Supplementary material for: The Usability of a Smartphone-Based Fall Risk Assessment App for Adult Wheelchair Users: Observational Study
Source: JMIR Form Res. 2022 Sep 16;6(9):e32453. doi: 10.2196/32453 (PMC9526126; doi:10.2196/32453)
Supplement: Multimedia Appendix 1 [file formative_v6i9e32453_app1.docx]

### Multimedia Appendix 1

**Instructional Prompt:**

*“Next, we will complete the fall risk health application, Steady-Wheels. The Steady-Wheels application will provide visual and auditory instructions to lead you through a series of seated balance tasks. While going through the application I would like you to say your thought processes out loud. Please focusing on continued talking and communication. This is to provide the researchers with clear insight into your view of the health app. I would like you to go through the steps of this application as independently as possible, but I am here if you get stuck along the way. Do you have any questions?”*
